# The Effects of Statins on Cognitive Performance Are Mediated by Low-Density Lipoprotein, C-Reactive Protein, and Blood Glucose Concentrations

**DOI:** 10.1093/gerona/glad163

**Published:** 2023-07-11

**Authors:** Mélissa Gentreau, Gull Rukh, Maud Miguet, Laura E Clemensson, Ahmed M Alsehli, Olga E Titova, Helgi B Schiöth

**Affiliations:** Functional Pharmacology and Neuroscience, Department of Surgical Sciences, University of Uppsala, Uppsala, Sweden; Functional Pharmacology and Neuroscience, Department of Surgical Sciences, University of Uppsala, Uppsala, Sweden; Functional Pharmacology and Neuroscience, Department of Surgical Sciences, University of Uppsala, Uppsala, Sweden; Functional Pharmacology and Neuroscience, Department of Surgical Sciences, University of Uppsala, Uppsala, Sweden; Department of Physiology, Faculty of Medicine, King Abdulaziz University, Jeddah, Kingdom of Saudi Arabia; Medical Epidemiology, Department of Surgical Sciences, University of Uppsala, Uppsala, Sweden; Functional Pharmacology and Neuroscience, Department of Surgical Sciences, University of Uppsala, Uppsala, Sweden

**Keywords:** Biomarkers, Cholesterol, Cognition, Hydroxymethylglutaryl CoA reductase inhibitors, Inflammation

## Abstract

Statins are widely used for cardiovascular disease prevention but their effects on cognition remain unclear. Statins reduce cholesterol concentration and have been suggested to provide both beneficial and detrimental effects. Our aim was to investigate the cross-sectional and longitudinal association between statin use and cognitive performance, and whether blood low-density lipoprotein, high-density lipoprotein, triglycerides, glucose, C-reactive protein, and vitamin D biomarkers mediated this association. We used participants from the UK biobank aged 40–69 without neurological and psychiatric disorders (*n* = 147 502 and *n* = 24 355, respectively). We performed linear regression to evaluate the association between statin use and cognitive performance and, mediation analysis to quantify the total, direct, indirect effects and the proportion meditated by blood biomarkers. Statin use was associated with lower cognitive performance at baseline (β = −0.40 [−0.53, −0.28], *p* = <.0001), and this association was mediated by low-density lipoprotein (proportion mediated = 51.4%, *p* = .002), C-reactive protein (proportion mediated = −11%, *p* = .006) and blood glucose (proportion mediated = 2.6%, *p* = .018) concentrations. However, statin use was not associated with cognitive performance, measured 8 years later (β = −0.003 [−0.11, 0.10], *p* = .96). Our findings suggest that statins are associated with lower short-term cognitive performance by lowering low-density lipoprotein and raising blood glucose concentrations, and better performance by lowering C-reactive protein concentrations. In contrast, statins have no effect on long-term cognition and remain beneficial in reducing cardiovascular risk factors.

Statins are among the most widely prescribed medications for primary and secondary prevention of cardiovascular diseases (1). They are 3-hydroxy-3-methylglutaryl coenzyme A reductase (HMGCR) inhibitors that lead to reduced blood cholesterol concentration (2). High blood cholesterol at midlife has been associated with cognitive decline that may lead to the development of Alzheimer’s disease (AD) and dementia (3,4). Thus, the period, where cognitive dysfunction is manifesting but dementia diagnosis is not established, may offer a potential therapeutic window for interventions based on repurposing of statins (5). Besides, statins might have effects beyond lipid lowering such as anti-inflammatory and anti-oxidant effects that may prevent cognitive dysfunction (6). However, the FDA has reported short-term cognitive dysfunction in the form of memory loss and confusion, now listed as possible adverse effects, raising the question of the beneficial effect of statins in preventing cognitive dysfunction (7).

Indeed, randomized controlled trials (RCTs) and observational studies have reported conflicting results that can be explained by short follow-ups, low sample sizes, various designs, or reverse causation (8). For example, the Sydney Memory and Ageing and the Alzheimer’s Disease Neuroimaging Initiative (ADNI) studies did not show a significant association between statin use and cognitive decline (9,10) but another study found that statin use was associated with slower cognitive decline over 10-year follow-up (11). Hence, the effects of statins on cognition are still debated and need to be replicated.

In addition, the cognitive effects of statins may differ depending on statin- and individual-related factors such as statin lipophilicity, sex, ethnicity, comorbidity, and genetic factors (12). For instance, lipophilic statins may have a greater effect on cognition as they easily cross the blood-brain barrier (13), and a recent meta-analysis shows that hydrophilic statins lowered the risk of dementia more strongly than lipophilic statins (14). Regarding, individual-related factors, a recent study showed that sex and ethnicity modify the association between statins and AD risk: high statin exposure was associated with lower AD risk for everyone except Black men (15). Lower cognitive decline was also demonstrated in statin users with a history of coronary heart disease (CHD) compared with nonusers (9), and statins may be more effective in apolipoprotein e ε4 allele (APOE4) carriers, as this is the major genetic risk factor for AD and is associated with higher cholesterol concentration (11).

Finally, statins may exert both negative and positive effects that could explain the disparity between studies. Indeed, previous research has demonstrated that statins can affect β-cell function and insulin sensitivity (16), and epidemiological studies have shown that statin use was associated with increased fasting blood glucose and diabetes progression (17,18). Independently of statin use, poor glycemic control, and diabetes are well-established risk factors for cognitive decline and AD (19). Therefore, statins might contribute to cognitive dysfunction through blood glucose dysregulation.

Nevertheless, statins also demonstrated anti-inflammatory effects. Clinical trials have consistently shown that statins lower C-reactive protein (CRP) concentrations, a marker of inflammation, by 15%–30%, independently of their effect on cholesterol (20). Moreover, inflammation has been linked to cognitive decline (21) suggesting that the anti-inflammatory properties of statins could be beneficial for cognitive function.

Another interesting aspect of statin use is its potential anti-oxidant effects. Some studies have suggested that statins, particularly simvastatin, and atorvastatin, may increase blood vitamin D concentrations. These statins are metabolized by the same cytochrome P450 enzyme (CYP3A4) as vitamin D (22), which could explain this interaction. Besides, higher vitamin D concentrations have been associated with better cognitive function (23). Therefore, statin could contribute to protecting cognition through these anti-oxidant effects.

Yet, no study has investigated whether diabetogenic, anti-inflammatory, and/or anti-oxidant effects mediated the relationship between statin use and cognition. Therefore, we ascertained whether statin use is associated with cognitive performance in short- and long-term. Then, because the primary effect of statin is to reduce blood cholesterol concentration, we first investigated whether the association between statin use and cognitive performance is mediated by low-density lipoprotein (LDL) and high-density lipoprotein (HDL) concentrations. Second, we asked whether this association is also mediated by diabetogenic, anti-inflammatory, and/or anti-oxidant effects using triglycerides, glucose, CRP, and vitamin D blood biomarkers.

## Method

### Study Population

**Figure 1.**
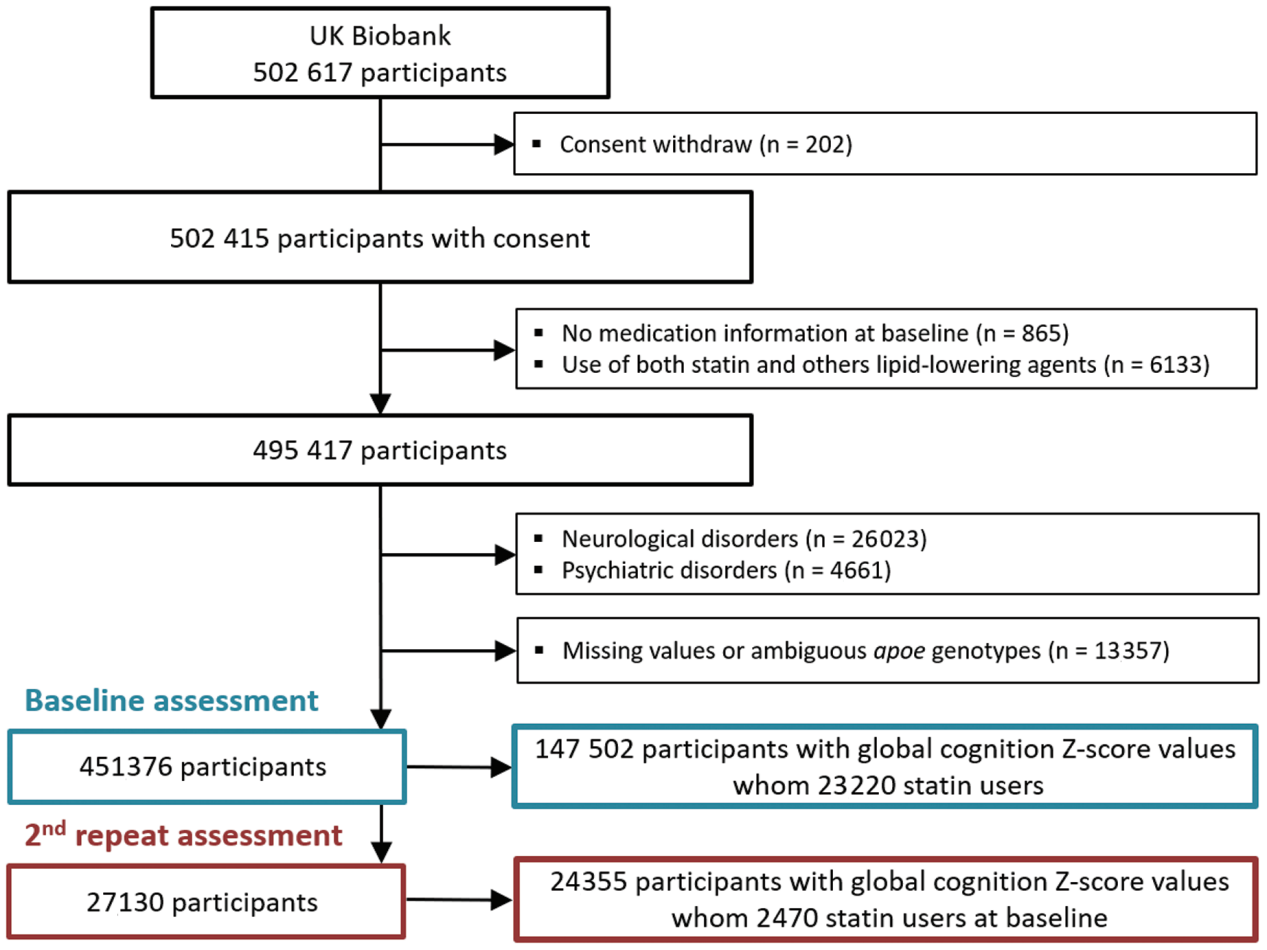
Flow chart of participants’ selection at baseline (2006–2010) and the second repeat assessment (2014–2017).

The UK Biobank is a prospective cohort study of approximately 500 000 participants aged 40–69 from England, Scotland, and Wales recruited between 2006 and 2010. A description of the study has been provided elsewhere (24) and detailed information is available on the UK Biobank website (www.ukbiobank.ac.uk). Baseline data collection included sociodemographic and lifestyle information, medical history, medication use, physical measurements, and biological samples. The first repeat assessment (2012–2013) included ~20 000 participants who lived near an assessment center. The second repeat assessment (2014+) is still ongoing. The North West Multi-Centre Research Ethics Committee approved the UK Biobank study protocol and the Regional Ethics Committee of Uppsala (Sweden) further approved the data use. All participants provided written informed consent. We used data released in January 2019. The present study excluded participants who withdrew their consent (*n* = 202), did not give medication information at baseline (*n* = 865), used both statins and other lipid-lowering medications (*n* = 6133), had prevalent neurological (*n* = 26 023) or psychiatric disorders (*n* = 4661) at baseline, and had missing values or ambiguous *APOE* genotypes (*n* = 13 357). The cross-sectional and longitudinal analyses included participants who had no missing data for the global cognition *Z*-score at baseline (*n* = 147 502) and at the second repeat assessment (*n* = 24 355), respectively (Figure 1).

### Statin Use

Information regarding regular cholesterol-lowering medications use was collected through a touchscreen questionnaire at baseline, verified during verbal interview, and reported as medication codes. Medication codes corresponding to statins were identified (Supplementary Appendix A) and summarized in a single variable (statin use: yes/no). A similar procedure was performed for collecting information regarding use of non-statin cholesterol-lowering medications (Supplementary Appendix B).

### Blood Samples

Nonfasting blood samples were collected at baseline. The protocol for the collection, processing, and archiving of blood samples has been previously described (25). LDL, HDL, triglycerides, and blood glucose concentrations were quantified using routine enzymatic methods. Serum CRP concentration was measured by immunoturbidimetry and the data were log-transformed for the analysis. Serum vitamin D concentration was quantified using a chemiluminescent immunoassay. Details about assay methods and quality control procedures are described elsewhere (26,27).

### Cognitive Tests

Participants completed 4 cognitive tests on the touchscreen questionnaire evaluating different cognitive domains (Supplementary Material): visuospatial memory (pairs matching test), reasoning (fluid intelligence test), processing speed (reaction time test), and prospective memory (prospective memory test). From these 4 cognitive test scores, we created a global cognitive *Z*-score as a proxy for global cognition. First, the 4 cognitive scores were re-coded such that higher positive values correspond to better cognitive performance. Then, we calculated *Z*-score for each cognitive domain by centering by the mean and reducing by the standard deviation. Finally, *Z*-scores were summed together to represent the global cognitive *Z*-score. The same procedure was applied to the cognitive scores of the second repeat assessment used for the longitudinal analysis.

### APOE Genotyping

Genotyping was performed using the Affymetrix UK Biobank and UK BiLEVE Axiom array (28,29). APOE genotypes were determined based on the 2 SNPs, rs429358 and rs7412 (Appendix C) as described elsewhere (30). Participants heterozygous for both SNPs resulted in ambiguous genotypes (ε2/ε4 or ε1/ε3) that did not clearly identify them as carriers of the ε4 allele. They were, therefore, excluded (31). APOE4 status was defined as carrying at least 1 ε4 allele.

### Covariates

At the baseline assessment, general information was collected on the touchscreen questionnaire. Ethnicity was recoded as white (British, Irish, or any other white background) versus non-white (ie, Mixed, Asian, or Asian British, Black or Black British, Chinese and another ethnic group) due to large majority of participants self-reporting as white (32). Education level was recoded from the education qualifications data as achieving college or university degree, as previously described (32,33). Townsend deprivation index (TDI) was used as a proxy of socioeconomic status of the participant based on the area of residence at baseline. Alcohol use and smoking status were defined as never, past, or current. Body mass index (BMI) was calculated from measured weight/height² (kg/m²). Physical activity was defined as number of days per week of moderate activity and was recoded into categories of low (0–1), medium (2–4), and high (5–7). History of diabetes, stroke, and CHD (angina and/or heart attack) were self-reported.

### Statistical Analysis

#### Association between statin use and cognition

A linear regression model was used to evaluate the association between statin use at baseline and global cognition, either at baseline (cross-sectional analysis) or at the second repeat assessment (longitudinal analysis). The covariates considered as confounders, (selected using Direct Acyclic Graph method and based on literature data (34)) included assessment center, age, sex, education, APOE4 status, TDI, ethnicity, alcohol use, smoking, BMI, physical activity, a history of diabetes, stroke, CHD, and hypertension (Supplementary Figure 1). Interaction between statin and confounders were tested and kept in the final model when statistically significant (ie, statin × sex, statin × ethnicity, statin × diabetes, statin × hypertension, and statin × CHD). Indeed, participants with history of diabetes, stroke, CHD, or hypertension are more likely to be prescribed statins (1) and are at risk of cognitive dysfunction (35). We repeated this analysis for each cognitive test. Pairs matching score was modeled using generalized linear model with a negative binomial distribution; fluid intelligence score and reaction time were modeled using linear model; prospective memory score was modeled using a logistic model, according to their respective distribution (Supplementary Figure 2).

Because of missing values for blood biomarkers (LDL, 5.5%; HDL 13.8%; triglycerides, 5.4%; blood glucose, 13.8%; CRP, 5.5%; vitamin, D 9.5%), the sample size varies across mediation analyses.

#### Mediation analysis

**Figure 2.**
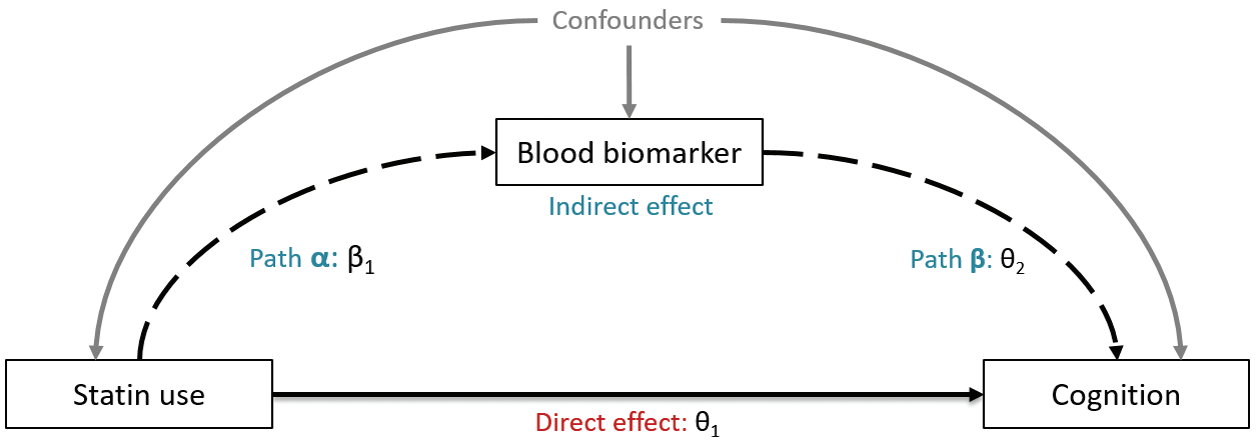
Mediation analysis model of the associations between statin use and cognition, mediated by blood biomarkers independently (ie, LDL, HDL, triglycerides, blood glucose, CRP, and vitamin D). Path α represents the regression coefficient β_1_ for the association between statin use and the blood biomarker. Path β represents the regression coefficient θ_2_ for the association between the blood biomarker and the cognition. The indirect effect is represented by dotted arrows and the direct effect is by continuous arrow. The total effect is the sum of the direct and indirect effect.

We performed a mediation analysis based on the counterfactual outcome framework (36–38) to assess the mediation of blood biomarkers in the association between statin use and cognition. We considered each blood biomarker individually. The total effect of statin use on cognition was decomposed into direct and indirect effects by fitting two models: a mediator and an outcome model. The mediator model estimates the association between statin use and a blood biomarker (path α, Figure 2). The outcome model estimates the association between a blood biomarker and cognition, adjusted for statin use (path β, Figure 2). The average direct (θ_1_), indirect (β_1_θ_2_), total effects (θ_1_+ β_1_θ_2_), and their 95% confidence intervals were computed with the R *mediation* package (39) using 1000 simulations. The proportion mediated was calculated as (indirect effect/total effect) (38). We checked that there was no significant interaction between the exposure and the mediator (ie, statin use and blood biomarker) and considered as identifying assumptions for the interpretation of the average total effect, direct effect, indirect effect, and the proportion mediated, that there was no unmeasured confounding.

#### Sensitivity analyses

Mediation analysis was repeated (a) excluding participants with a history of heart disease, stroke, diabetes, and hypertension to confirm that the mediation analysis results were not due to residual confounding bias; (b) restricting the analysis to simvastatin users versus nonusers, as most of statin users took simvastatin.

All statistical analyses were performed using R version 4.1.3 (The R Foundation for Statistical Computing, Vienna, Austria; www.R-project.org).

## Results

The sample of the cross-sectional analysis included 23 220 statin users and 124 282 nonusers. The participants’ mean age was 56.59 (*SD* = 8.17) years and 45.68% were men (Table 1). Compared with nonusers, statin users were older and more likely to be men. They were less likely to have a college or university degree and more likely to carry APOE4. They tended to have a higher BMI and more often reported a history of diabetes, stroke, CHD, or hypertension. Most statin users took simvastatin (74.50%).

**Table 1.** Characteristics of the Study Sample at Baseline

Characteristics Mean (*SD*) or *n* (%)	All Sample *n* = 147 502	Statin Nonusers *n* = 124 282	Statin Users *n* = 23 220
Age	56.59 (8.20)	55.66 (8.20)	61.52 (6.04)
Sex (men)	67 385 (45.68)	52 907 (42.57)	14 478 (62.35)
College or university degree	51 284 (34.77)	44 977 (36.19)	6307 (27.16)
TDI	−1.22 (2.89)	−1.26 (2.87)	−0.99 (3.02)
Ethnic background			
White	136 442 (92.50)	115 093 (92.61)	21 349 (91.94)
Mixed	1081 (0.73)	954 (0.77)	127 (0.55)
Asian or Asian British	3962 (2.69)	3069 (2.47)	893 (3.85)
Black and Black British	3356 (2.28)	2946 (2.37)	410 (1.77)
Chinese	538 (0.36)	473 (0.38)	65 (0.28)
Other ethnic group	1571 (1.07)	1303 (1.05)	268 (1.15)
APOE4 carriers	37 608 (25.50)	30 777 (24.76)	6831 (29.42)
Alcohol use			
Never	6277 (4.26)	5115 (4.12)	1162 (5)
Previous	4961 (3.36)	3918 (3.15)	1043 (4.49)
Current	136 135 (92.29)	115 147 (92.65)	20 988 (90.39)
Smoking status			
Never	81 496 (55.25)	70 888 (57.04)	10 608 (45.68)
Previous	51 269 (34.76)	41099 (33.07)	10 170 (43.80)
Current	14 281 (9.68)	11 942 (9.61)	2339 (10.07)
BMI (kg/m²)	27.28 (4.72)	26.94 (4.61)	29.15 (4.87)
Physical activity (days/week)			
0–1	26 916 (18.25)	22 350 (17.98)	4566 (19.66)
2–4	56 380 (38.22)	47 725 (38.40)	8655 (37.27)
5–7	57 656 (39.09)	49 071 (39.48)	8585 (36.97)
Diabetes	6027 (4.09)	1736 (1.40)	4291 (18.48)
Stroke	1430 (0.97)	472 (0.38)	958 (4.13)
CHD	5670 (3.84)	1197 (0.96)	4473 (19.26)
Hypertension	37 000 (25.08)	23 235 (18.70)	13 765 (59.28)
Type of statin			
No statin	124 282 (84.26)		–
Pravastatin	692 (0.47)		692 (2.98)
Rosuvastatin	742 (0.50)		742 (3.20)
Atorvastatin	4433 (3.01)		4433 (19.09)
Fluvastatin	53 (0.04)		53 (0.23)
Simvastatin	17 300 (11.73)		17 300 (74.50)
LDL (mmol/L)	3.56 (0.86)	3.71 (0.81)	2.77 (0.68)
HDL (mmol/L)	1.47 (0.39)	1.50 (0.39)	1.32 (0.35)
Triglycerides (mmol/L)	1.71 (0.99)	1.67 (0.98)	1.89 (1.05)
Blood glucose (mmol/L)	5.18 (1.11)	5.09 (0.87)	5.65 (1.86)
CRP (mg/L)	2.48 (4.17)	2.46 (4.14)	2.64 (4.37)
Vitamin D (nmol/L)	50.43 (21.28)	50.37 (21.18)	50.69 (21.77)

*Notes*: APOE4 = apolipoprotein E ε4; BMI = body mass index; CHD = coronary heart disease; CRP = C-reactive protein; *SD* = standard deviation; HDL = high-density lipoprotein; LDL = low-density lipoprotein; TDI = Townsend Deprivation Index.

The sample of the longitudinal analysis included 2470 statin users and 21 885 nonusers. The mean follow-up time between the baseline and the second repeat assessment was 8.0 (*SD* = 1.5) years. The participants’ mean age was 54.68 (*SD* = 7.45) years, 48.80% were men. The characteristics between statin users and nonusers were comparable to the sample of the cross-sectional analysis (Supplementary Table 1).

Participants with missing data did not differ from the analytical sample.

### Cross-Sectional Association Between Statin Use and Cognitive Performance

First, we assessed the association between statin use and cognition at baseline without taking into account mediator paths. Statin use was significantly associated with lower global cognitive performance (β = −.403 [−0.531, −0.276], *p* = <.0001) after adjustments for assessment center, age, sex, education, APOE4 status, TDI, ethnicity, alcohol use, smoking, BMI, physical activity, history of diabetes, stroke, hypertension, CHD, and considering interactions between statin and sex, ethnicity, diabetes, hypertension, or CHD (Supplementary Table 2). The predicted values of the global cognition *Z*-score in the function of statin use and confounder interactions are presented in Supplementary Figure 3.

For the individual cognitive tests, we found similar results regarding fluid intelligence and reaction time tests (Supplementary Table 2). Statin use was associated with the lower fluid intelligence score (β = −0.231 [−0.349, −0.114], *p* = .0001) and higher reaction time (β = 12.993 ms [8.457, 17.529], *p* < .0001). However, no significant association was found regarding pairs matching and prospective memory scores.

### Longitudinal Association Between Statin Use and Cognitive Performance

Second, we evaluated the association between statin use and cognition at the second repeat assessment. Statin use was not associated with global cognitive performance measured 8 years later after adjustments for confounders (β = −0.003 [−0.109, 0.104], *p* = .96) and, was not associated with any of the 4 cognitive scores (Supplementary Table 3).

### Mediation of Blood Biomarkers in the Association Between Statin Use and Cognitive Performance

Third, we investigated whether and how much LDL, HDL, triglycerides, blood glucose, CRP, and vitamin D concentrations mediated the association between statin use and cognitive performance (Figure 2). Because no association between statin use and cognition was found in the longitudinal analysis, the mediation analysis was performed regarding cognitive performance at baseline.

We assessed the association between statin use and each blood biomarker (path α, Figure 2), and each blood biomarker and global cognition at baseline (path β, Figure 2). The path α models showed that statin use was associated with lower LDL, HDL, and CRP concentrations and higher triglycerides, blood glucose and, vitamin D concentrations. Moreover, the path β models showed that higher LDL, HDL, and triglycerides and, lower CRP, blood glucose, and vitamin D concentrations were associated with increased global cognitive performance (Table 2).

**Table 2.** Association Between Statin Use, Blood Biomarkers and Global Cognitive Function

Mediator	Path	β (CI)	*p* Value
Statin → LDL	Α	−0.642 (−0.690, −0.594)	<.0001
LDL → cognition	Β	0.043 (0.028, 0.059)	<.0001
Statin → HDL	Α	−0.083 (−0.105, −0.062)	<.0001
HDL → cognition	Β	0.041 (0.004, 0.077)	.0301
Statin → triglycerides	Α	0.092 (0.034, 0.15)	.002
Triglycerides → cognition	Β	0.018 (0.006, 0.03)	.0038
Statin → blood glucose	Α	0.086 (0.021, 0.152)	.0098
Blood glucose → cognition	Β	−0.018 (−0.030, −0.006)	.0033
Statin → CRP	Α	−0.029 (−0.045, −0.014)	.0004
CRP → cognition	Β	−0.162 (−0.208, −0.116)	<.0001
Statin → Vitamin D	Α	1.972 (0.703, 3.241)	.0023
Vitamin D → cognition	Β	−0.001 (−0.001, −2 × 10^−4^)	.007

*Notes*: Path α represents the regression coefficient for the association between statin use and the blood biomarker. Path β represents the regression coefficient for the association between the blood biomarker and cognition. CI = confidence interval; CRP = C-reactive protein; HDL = high-density lipoprotein; LDL = low-density lipoprotein.

Then, the average total, direct, indirect effects, and the proportion mediated were calculated (Table 3). The effect of statin on global cognitive performance (lower score) was mediated by decreased LDL (51.38%), HDL (4.35%), increased blood glucose (2.64%), and vitamin D (2.38%) concentrations. However, increased triglycerides concentration and decreased CRP concentration attenuated the effects of statins on global cognition with a negative mediated proportion of −0.53% and −11.02%, respectively.

**Table 3.** The Mediation Effect of Blood Biomarkers on the Cross-Sectional Association Between Statin Use and Global Cognition.

Mediator	*n*	Total Effect β (CI)	Direct Effect β (CI)	Indirect Effect β (CI)	Proportion Mediated	*P* value of the Mediated Effect
LDL	130 707	−0.067 (−0.124, −0.007)	−0.032 (−0.076, 0.013)	−0.034 (−0.048, −0.021)	51.38%	.002
HDL	119 244	−0.061 (−0.112, −0.01)	−0.058 (−0.107, −0.010)	−0.003 (−0.005, −0.0003)	4.35%	.04
Triglycerides	130 886	−0.067 (−0.113, −0.015)	−0.067 (−0.113, −0.016)	4 × 10^−4^ (2 × 10^−5^, 0.001)	−0.53%	.042
Blood glucose	119 136	−0.062 (−0.114, −0.011)	−0.060 (−0.111, −0.010)	−0.002 (−0.003, −3 × 10^−4^)	2.64%	.018
CRP	130 710	−0.065 (−0.114, −0.018)	−0.072 (−0.119, −0.028)	0.007 (0.005, 0.010)	−11.02%	.006
Vitamin D	125147	−0.064 (−0.113, −0.015)	−0.063 (−0.110, −0.015)	−0.002 (−0.003, −4 × 10^−4^)	2.38%	.01

*Notes*: CI = confidence interval; CRP = C-reactive protein; HDL = high-density lipoprotein; LDL = low-density lipoprotein.

### Sensitivity Analyses

When we excluded participants with the history of CHD, stroke, diabetes, and hypertension from the mediation analysis, LDL, triglycerides, and CRP still significantly mediated the cognitive effect of statin but HDL, blood glucose, and vitamin D did not (Supplementary Table 4).

Finally, the mediation analysis was performed on simvastatin users versus nonusers. We found that the cognitive effect of simvastatin was mediated by decreased LDL (41.33%) and CRP (−8.60%), increased blood glucose (1.94%), and vitamin D (1.93%). However, no significant mediation by HDL and triglycerides concentrations were found (Supplementary Table 5).

## Discussion

We find statin use is associated with lower performance in global cognition, reasoning, and processing speed at baseline although this association is weakened in men, white ethnicity, and participants with history of diabetes, CHD, or hypertension. In addition, the effects of statins on global cognition were mediated by a decrease in LDL, CRP, and an increase in blood glucose concentrations. About 51.38% of the effects of statins were mediated by LDL and 2.64% by blood glucose concentrations. The proportion mediated by CRP was −11.02%. In contrast, we did not find a significant association between statin use and cognition measured 8 years later. Overall, our findings suggest that statins are associated with negative effects on short-term cognition through lowering LDL and elevating blood glucose, and positive effects through lowering CRP.

We found that statin use was significantly associated with short-term cognitive dysfunction but not with long-term cognition. Previous studies on the effects of statins on cognition have yielded contradictory results. Regarding short-term cognition, some studies reported adverse effects. In the ASPREE cohort, statin users had significantly lower performance in global cognition and episodic memory (40). In a RCT, patients also demonstrated lower cognitive performance after 6 months of simvastatin therapy (41). However, in a study of 7191 statin users and 17 404 statin nonusers, statin was not significantly associated with cognitive impairment in the multivariate analysis (42). Regarding long-term cognition, there are mixed findings as recent observational studies with large sample sizes did not identify a significant association between statin use and cognitive decline (9,10,40), although some other studies found a protective association (11,43). These disparities between studies can be explained by different designs and adjustments. Indeed, the probability of statin use is conditioned by the presence of cardiovascular risk factors such as a history of hypertension, stroke, CHD, type 2 diabetes, or BMI, but these risk factors also affect cognition (1,35). Thus, by not adjusting for these factors, we might find an association between statin use and cognition that in fact, reflects the cardiovascular prevention by statins on cognition. We adjusted for cardiovascular risk factors and found significant protective interactions between statins and history of diabetes, CHD, and hypertension acknowledging the cardiovascular benefits that help prevent cognitive dysfunction. This protective effect was also found in older adults with type 2 diabetes (44), CHD, and hypertension (9). Therefore, although statins appear to exert short-term cognitive dysfunction, long-term statin use could outweigh these adverse effects by preventing cardiovascular diseases, which are important risk factors for cognitive decline and AD onset.

The mechanisms by which statins exert their cognitive effects are not fully elucidated. Here, we evaluated the mediating effects of diabetogenic, anti-inflammatory, and/or anti-oxidant blood biomarkers in the relationship between statin and cognition. On one hand, our results suggest that statins are associated with negative effects on cognition. We demonstrated that LDL mediated the association between statin use and cognition by 51.38%, suggesting that reduced LDL concentration may be detrimental to cognition. One explanation for this result might be that LDL and cognition have a U-shaped relationship. Indeed, it has been shown that individuals under 70 had reduced cognitive performance for both low and high LDL concentrations (45). Besides, cholesterol is an important component of brain function, involved in synaptic plasticity and transmission (46). Thus, a optimum level may be needed for good neuronal functioning. In addition, we found that blood glucose may also partially mediate the effects of statins on cognition, even when controlling for diabetes. Indeed, poor glycemic control and type 2 diabetes are known risk factors for cognitive decline and AD (19) and statin use has been associated with increased fasting blood glucose (17) and diabetes progression (18). Preclinical studies indicate that statins alter β-cell function by the inhibition of L-type calcium channels and decrease insulin sensitivity through reduced phosphorylation of the insulin receptor substrate-1 (IRS-1) (16). Thus, statins may contribute to cognitive dysfunction by drastically lowering LDL concentration and affecting glycemic control.

On the other hand, we show that statins are associated with positive effects on cognition through lowering CRP. It has been suggested that simvastatin inhibits CRP by decreasing mevalonate and geranylgeranyl pyrophosphate (GGPP), products of the cholesterol biosynthesis pathway (mevalonate pathway) (47). Statins have also been associated with the reduction of other inflammatory markers such as TNF-α, IL-6, IL-1, (48) and inflammation has been associated with cognitive decline (21). Thus, statin may exert anti-inflammatory effects that help protect cognition.

Finally, we found inconsistent results regarding the mediation by triglycerides, HDL, and vitamin D. First, the mediations by HDL and triglycerides were not robust as the mediated proportions were no longer significant in the analysis on simvastatin use, suggesting either divergence effects depending on statin type or chance findings. Besides, in our sample, statin users had higher triglyceride and lower HDL concentrations than nonusers although RCTs demonstrated opposite results (49). Second, high vitamin D status has been found to be protective for cognition (23) but we found the opposite effect. These results may be explained by the cross-sectional design of our study, which does not rule out reverse causality. However, several interventional studies reported that statins may increase vitamin D concentration (50–52). In addition, other studies suggest that vitamin D status strengthens the effects of statins and may have a synergic action by potentiating the reduction of LDL and triglyceride concentrations (53,54). Thus, statins may promote or potentiate anti-oxidant effects through vitamin D concentration.

The major strengths of this study include the large number of participants to detect interaction effects and small effect sizes, as well as the longitudinal design. However, the follow-up period may not be long enough to detect an association between statin use and long-term cognition. This may also be due to a lack of statistical power given the reduced sample size in the longitudinal analysis; or selection bias, as the participants completing the cognitive tests at the second repeat assessment were slightly younger, more likely to have achieved college or university degree and less likely to have a medical history; or simply because participants usually score better the second time. In addition, cognitive dysfunction may result from different conditions, not all of which will manifest in future dementia, which may explain the difference in studies evaluating the benefit of statins in AD onset (8). This study is the first to quantify the mediation by blood biomarkers in the association between statins and cognition. Besides, we adjusted for multiple confounders. However, our study presents some limitations. First, we could not adjust for the duration of statin therapy, dosage, or the age at which dyslipidemia was diagnosed because this information was not available in the UK Biobank. Second, for most participants, blood samples were not collected in the fasting state, which may alter estimates, although earlier studies found no clinical difference between nonfasting and fasting lipid concentrations (55). Regarding blood glucose concentrations, we adjusted for type 2 diabetes to attenuate this bias. Third, the mediation analysis is based on a cross-sectional design. Hence, these results should be interpreted with caution as they do not establish causality. However, our study does allow us to explore potential associations and generate hypotheses for further investigation.

## Conclusion

This study displays that statin use is associated with both positive and negative effects on cognitive performance which may explain why most longitudinal studies, including ours, have not found a significant association between statins and long-term cognition. This study provides an explanation for the conflicting results of statin effects on cognition and a foundation for further studies to understand the underlying mechanisms. Statin use remains effective in preventing cardiovascular disease which is an important component of cognitive decline and AD onset.

## Supplementary Material

glad163_suppl_Supplementary_Materials

## Data Availability

The data that support the findings of this study are available from the UK Biobank data resources but restrictions apply to the availability of these data, which were used under license for the current study, and so are not publicly available. Data are however available from the authors upon reasonable request and with permission of the UK Biobank data resources.
