# Supplementary material for: The Effects of Statins on Cognitive Performance Are Mediated by Low-Density Lipoprotein, C-Reactive Protein, and Blood Glucose Concentrations
Source: J Gerontol A Biol Sci Med Sci. 2023 Jul 11;78(11):1964–72. doi: 10.1093/gerona/glad163 (PMC10613010; doi:10.1093/gerona/glad163)
Supplement: glad163_suppl_Supplementary_Materials [file glad163_suppl_supplementary_materials.docx]

**Appendix A.** Statin medication codes

| **Type** | **Name** | **Data-coding** |
| --- | --- | --- |
| Simvastatins | Simvastatin | 1140861958 |
|  | Zocor | 1140881748 |
|  | Simvador | 1141188146 |
| Rosuvastatins | Rosuvastatin | 1141192410 |
|  | Crestor | 1141192414 |
| Pravastatins | Pravastatin | 1140888648 |
|  | Lipostat | 1140861970 |
| Fluvastatins | Fluvastatin | 1140888594 |
|  | Lescol | 1140864592 |
| Atorvastatins | Atorvastatin | 1141146234 |
|  | Lipitor | 1141146138 |

**Appendix B.** Non-statin cholesterol-lowering medication codes

| **Category** | **Type** | **Name** | **Data-coding** |
| --- | --- | --- | --- |
| Fibrates | Clofibrate | Clofibrate | 1140861944 |
|  |  | Atromid-s 500mg capsule | 1140861946 |
|  | Bezafibrate | Bezafibrate | 1140861924 |
|  |  | Bezalip 200mg tablet | 1140861926 |
|  |  | Bezalip-mono 400mg m/r tablet | 1140861928 |
|  | Gemfibrozil | Gemfibrozil | 1140861856 |
|  |  | Lopid 300 capsule | 1140861858 |
|  | Fenofibrate | Fenofibrate | 1140861954 |
|  | Ciprofibrate | Ciprofibrate | 1140862026 |
|  |  | Modalim | 1140862028 |
| Biles acid sequestrants | Colestyramine | Cholestyramine | 1140865576 |
|  |  | Cholestyramine product | 1141157416 |
|  |  | Cholestyramine+aspartame 4g/sachet powder | 1140861942 |
|  |  | Questran 4g/sachet powder | 1140861936 |
|  |  | Colestyramine | 1140909780 |
|  |  | Colestyramine+aspartame 4g/sachet powder | 1141180722 |
|  |  | Colestyramine product | 1141180734 |
|  | Colestipol | Colestipol | 1140888590 |
|  |  | Colestid 5g/sachet granules | 1140861848 |
| Nicotinic acid and derivatives | Nicotinic acid | Nicotinic acid product | 1140861868 |
|  | Acipimox | Acipimox | 1140861892 |
|  |  | Olbetam 250mg capsule | 1140861894 |
| Others lipid modifying agents | Omega-3 | Omega-3/fish oil supplement | 1193 |
|  |  | Maxepa 1g capsule | 1140861884 |
|  | Ezetimibe | Ezetimibe | 1141192736 |
|  |  | Ezetrol 10mg tablet | 1141192740 |

**Appendix C.** APOE genotypes based on the rs429358 and rs7412 SNPs

|  | | **rs429358** | | |
| --- | --- | --- | --- | --- |
|  |  | CC | CT | TT |
| **rs7412** | CC | ε4 ε4 | ε4 ε3 | ε3 ε3 |
|  | TC | ε1 ε4 | ε1 ε3 or ε2 ε4 | ε2 ε3 |
|  | TT | ε1 ε1 | ε1 ε2 | ε2 ε2 |

**Supplementary Methods**

*Cognitive tests*

Participants completed four cognitive tests on the touchscreen questionnaire.

The **Pairs matching** test evaluates visuospatial memory and attention. Six pairs of matching symbol cards are displayed for 5 seconds and participants were asked to memorize and recall the position of 6 pairs of cards.^1^ Then, the cards are turned face down and participants were asked to touch as many pairs as possible in the fewest tries. The score is the number of incorrect matches. Thus, a higher score corresponds to a lower cognitive performance.

The **Fluid intelligence** test evaluates reasoning. Participants were given two minutes to answer as many of the 13 questions as possible.^2^ The score is the number of correct answers. Thus, a higher score corresponds to a higher cognitive performance.

The **Reaction time** test evaluates simple processing speed. Participants were shown two cards at a time and were asked to answer as quickly as possible if the cards match by pressing a button.^3^ The score is the mean reaction time to correctly identify matches in milliseconds across the last 8 rounds. Thus, a higher score corresponds to a lower cognitive performance.

In the **Prospective memory** test, participants were asked to remember to touch the orange circle instead of the blue square, before beginning the cognitive tests.^4^ If participants did not recall this instruction and chose the orange circle, they were scored as 1, otherwise 0. Thus, a higher score corresponds to a lower cognitive performance.

Not all participants completed all cognitive tests (i.e. Pairs matching n = 437,539; Fluid intelligence n =149,003; Reaction time n = 446,752; Prospective memory n = 154117) resulting in various sample size for the analyses.

***
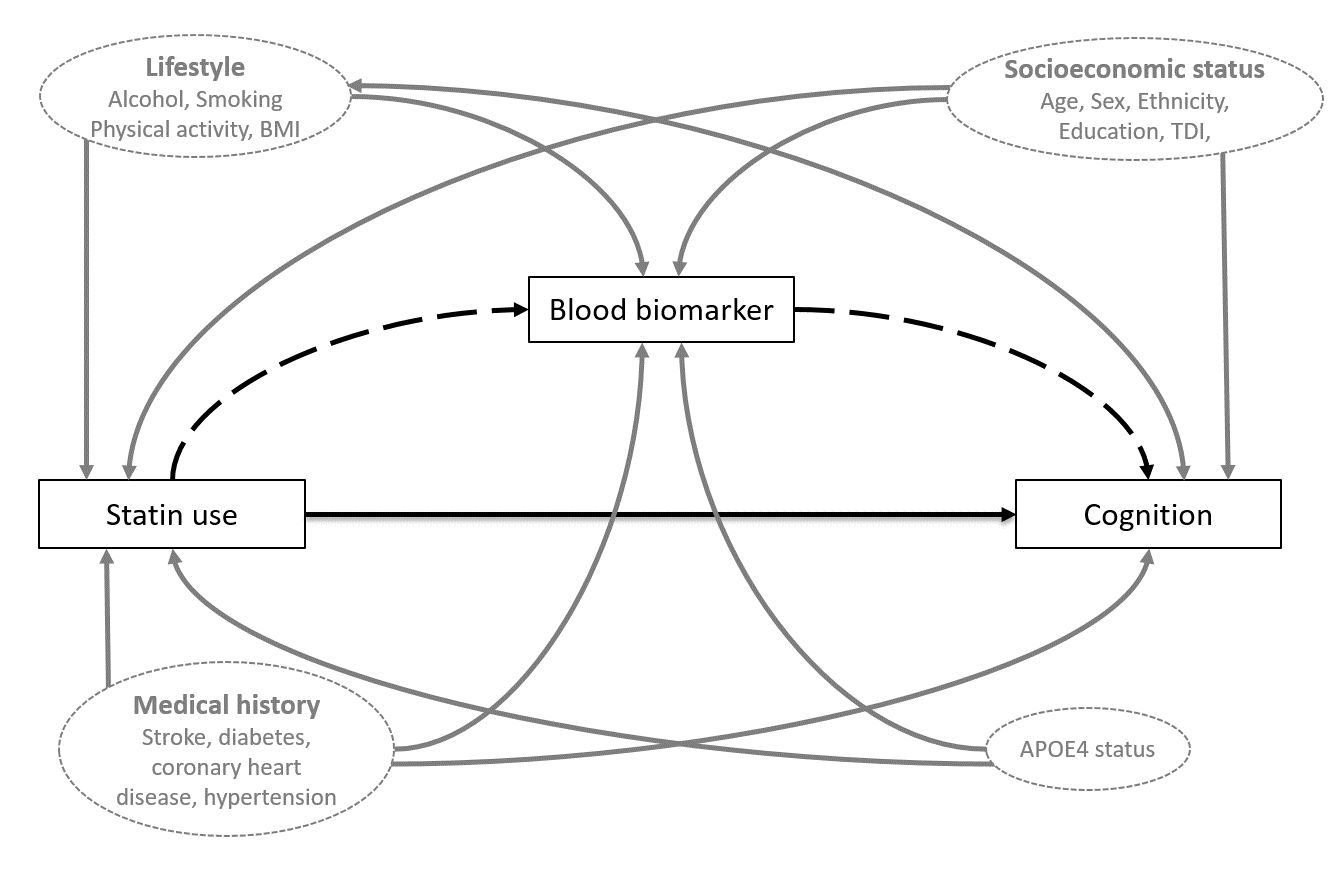
***

**Supplementary Figure 1.** Direct acyclic diagram graph of the relationships between, statin, blood biomarkers, cognition and potential confounders. Abbreviations: APOE4, Apolipoprotein E ε4, CHD, Coronary Heart Disease; CRP, C-Reactive Protein; SD, standard deviation; HDL, High-Density Lipoprotein; LDL, Low-Density Lipoprotein; TDI, Townsend Deprivation Index.


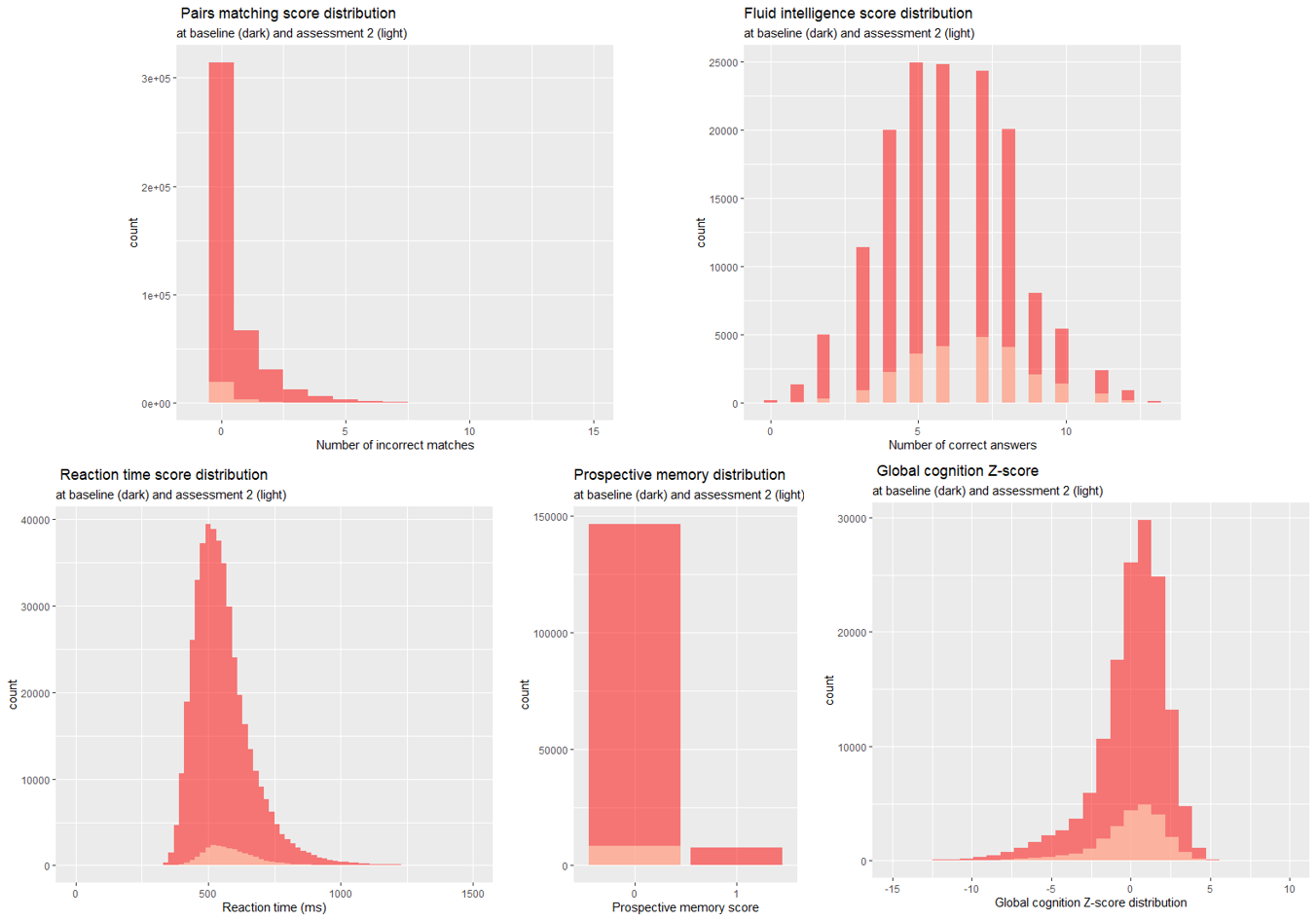


**Supplementary Figure 2.** Distribution of the four cognitive scores and the global cognition Z-score from baseline (dark) and second repeat assessment (light).

**Supplementary Table 1.** Baseline characteristics of the study sample at the second repeat assessment.

| **Characteristics**  Mean (SD) or n (%) | **All sample**  n = 24,355 | **Statin nonusers**  n = 21,885 | **Statin users**  n = 2,470 |
| --- | --- | --- | --- |
| Age | 54.68 (7.45) | 54.10 (7.39) | 59.79 (5.93) |
| Sex (Men) | 11885 (48.80) | 10141 (46.34) | 1744 (70.61) |
| College or university degree | 11144 (45.76) | 10171 (46.47) | 973 (39.39) |
| TDI | -2.04 (2.62) | -2.05 (2.61) | -2.01 (2.71) |
| Ethnic background |  |  |  |
| White | 23705 (97.33) | 21307 (97.36) | 2398 (97.09) |
| Mixed | 96 (0.39) | 87 (0.40) | 9 (0.36) |
| Asian or Asian British | 206 (0.85) | 171 (0.78) | 35 (1.42) |
| Black and Black British | 120 (0.49) | 115 (0.53) | 5 (0.20) |
| Chinese | 72 (0.30) | 69 (0.32) | 3 (0.12) |
| Other ethnic group | 82 (0.34) | 70 (0.32) | 12 (0.49) |
| APOE4 carriers | 6129 (25.17) | 5427 (24.8) | 702 (28.42) |
| Alcohol use |  |  |  |
| Never | 603 (2.48) | 544 (2.49) | 59 (2.39) |
| Previous | 541 (2.22) | 476 (2.18) | 65 (2.63) |
| Current | 23206 (95.28) | 20860 (95.32) | 2346 (94.98) |
| Smoking status |  |  |  |
| Never | 14863 (61.03) | 13619 (62.23) | 1244 (50.36) |
| Previous | 7956 (32.67) | 6902 (31.54) | 1054 (42.67) |
| Current | 1488 (6.11) | 1323 (6.05) | 165 (6.68) |
| BMI (kg/m²) | 26.65 (4.29) | 26.42 (4.20) | 28.66 (4.50) |
| Physical activity (days/week) |  |  |  |
| 0-1 | 5196 (21.33) | 4603 (21.03) | 593 (24.01) |
| 2-4 | 10339 (42.45) | 9343 (42.69) | 996 (40.32) |
| 5-7 | 8243 (33.85) | 7431 (33.95) | 812 (32.87) |
| Diabetes | 479 (1.97) | 143 (0.65) | 336 (13.60) |
| Stroke | 106 (0.44) | 39 (0.18) | 67 (2.71) |
| CHD | 515 (2.11) | 97 (0.44) | 418 (16.92) |
| Hypertension | 4521 (18.56) | 3130 (14.3) | 1391 (56.32) |
| Type of statin |  |  |  |
| No statin | 21885 (89.86) |  | - |
| Pravastatin | 64 (0.26) |  | 64 (2.59) |
| Rosuvastatin | 100 (0.41) |  | 100 (4.05) |
| Atorvastatin | 544 (2.23) |  | 544 (22.02) |
| Fluvastatin | 8 (0.03) |  | 8 (0.32) |
| Simvastatin | 1754 (7.20) |  | 1754 (71.01) |
| LDL (mmol/L) | 3.58 (0.83) | 3.67 (0.80) | 2.76 (0.66) |
| HDL (mmol/L) | 1.47 (0.37) | 1.48 (0.37) | 1.3 (0.32) |
| Triglycerides (mmol/L) | 1.66 (0.98) | 1.63 (0.97) | 1.84 (1.03) |
| Blood glucose (mmol/L) | 4.98 (0.95) | 4.94 (0.80) | 5.41 (1.71) |
| CRP (mg/L) | 2.07 (3.54) | 2.06 (3.51) | 2.21 (3.73) |
| Vitamin D (nmol/L) | 49.87 (20.85) | 49.66 (20.75) | 51.67 (21.6) |
| Follow-up time (years) | 7.97 (1.51) | 7.98 (1.51) | 7.81 (1.53) |

Abbreviations: APOE4, Apolipoprotein E ε4, CHD, Coronary Heart Disease; CRP, C-Reactive Protein; SD, standard deviation; HDL, High-Density Lipoprotein; LDL, Low-Density Lipoprotein; TDI, Townsend Deprivation Index.


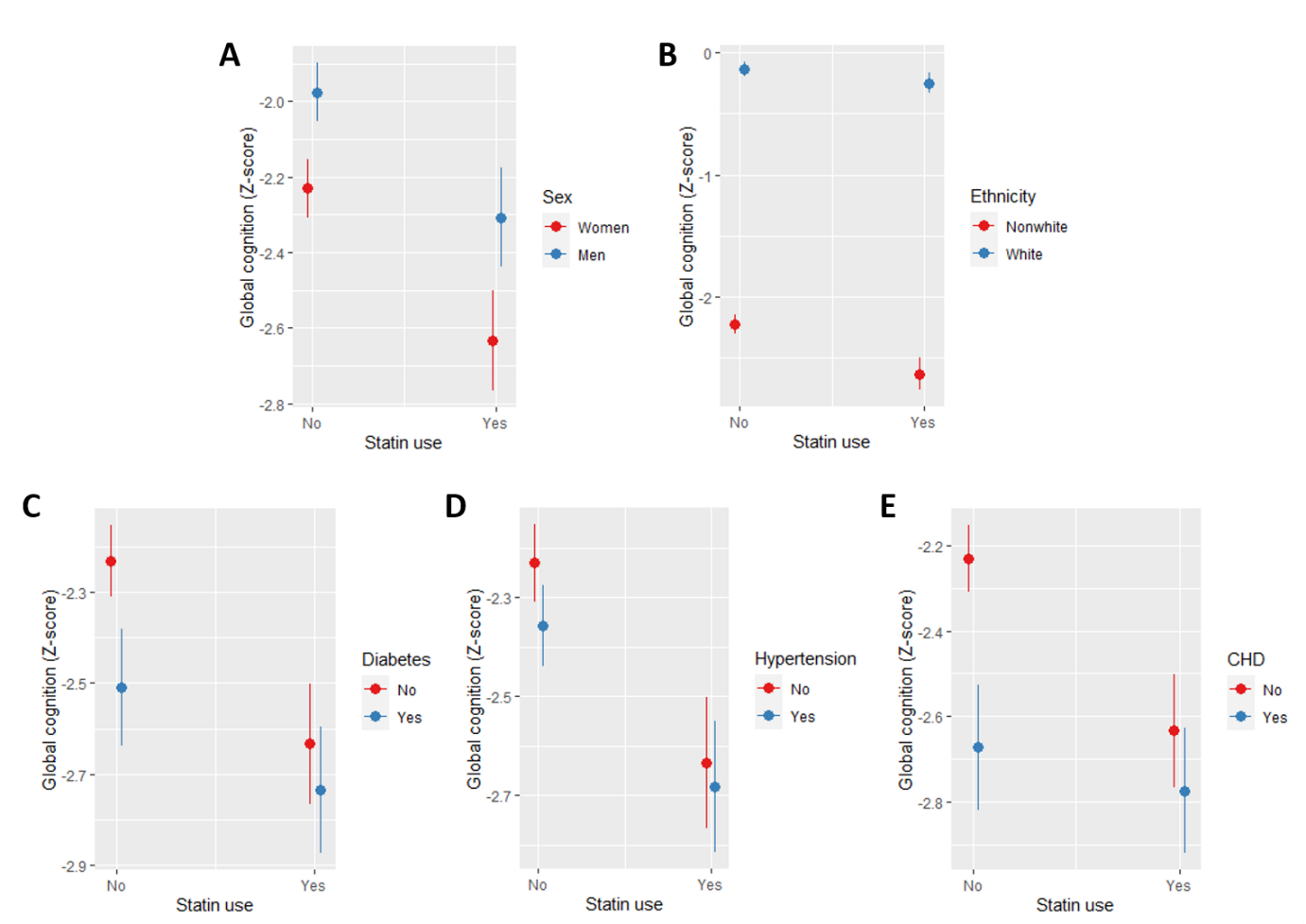


**Supplementary Figure 3.** Predicted values of global cognition Z-score at baseline in function of statin use according to different confounders: **(A)** women and men, **(B)** nonwhite and white ethnicity, **(C)** with and without diabetes, **(D)** with and without hypertension, **(E)** with and without CHD. Predictions were generated from the linear model adjusted for center, age, sex, education, APOE4 status, TDI, ethnicity, alcohol use, smoking, BMI, physical activity, history of diabetes, stroke, CHD and hypertension, and statin x confounder interactions (i.e., sex, ethnicity, diabetes, hypertension, and CHD). Abbreviation: APOE4, Apolipoprotein E ε4; BMI, Body Mass Index; CHD, Coronary Heart Disease; TDI, Townsend Deprivation Index.

**Supplementary Table 2.** Association between statin use and cognitive performance at **baseline** for each cognitive test.

| **Exposure** | **Z-score** ^2^ | | **Pairs matching** ^1^ | | **Fluid intelligence** ^2^ | | **Reaction time** ^2^ | | **Prospective memory** ^3^ | |
| --- | --- | --- | --- | --- | --- | --- | --- | --- | --- | --- |
|  | β (CI) | *P* value | $e^{\beta}$ (CI) | *P* value | β (CI) | *P* value | β (CI) | *P* value | OR (CI) | *P* value |
| Statin | -0.403  (-0.531, -0.276) | <0.0001 | 1.06  (0.98, 1.15) | 0.1286 | -0.231  (-0.349, -0.114) | 0.0001 | 12.993  (8.457, 17.529) | <0.0001 | 0.90  (0.75, 1.08) | 0.2417 |

Models were adjusted for assessment center, age, sex, education, APOE4 status, TDI, ethnicity, alcohol use, smoking, BMI, physical activity, history of diabetes, stroke, hypertension, CHD, statin x sex, statin x ethnicity, statin x diabetes, statin x hypertension, and statin x CHD.

^1^ Generalized linear model with negative binomial distribution; ^2^ Linear regression model; ^3^ Logistic regression model

Abbreviations: CHD, Coronary Heart Disease; CI, Confidence Interval; OR, odds ratio.

**Supplementary Table 3.** Association between statin use and cognitive performance at the **second repeat assessment** for each cognitive test.

| **Exposure** | **Z-score** ^2^ | | **Pairs matching** ^1^ | | **Fluid intelligence** ^2^ | | **Reaction time** ^2^ | | **Prospective memory** ^3^ | |
| --- | --- | --- | --- | --- | --- | --- | --- | --- | --- | --- |
|  | β (CI) | *P* value | $e^{\beta}$ (CI) | *P* value | β (CI) | *P* value | β (CI) | *P* value | OR (CI) | *P* value |
| Statin | -0.003  (-0.109, 0.104) | 0.9593 | 1.02  (0.91, 1.15) | 0.7359 | -0.036  (-0.132, 0.061) | 0.4711 | -1.030  (-6.112, 4.053) | 0.6913 | 1.04  (0.84, 1.29) | 0.7312 |

Models were adjusted for assessment center, age, sex, education, APOE4 status, TDI, ethnicity, alcohol use, smoking, BMI, physical activity, history of diabetes, stroke, hypertension, and CHD.

^1^ Generalized linear model with negative binomial distribution; ^2^ Linear regression model; ^3^ Logistic regression model

Abbreviations: CHD, Coronary Heart Disease; CI, Confidence Interval; OR, odds ratio.

**Supplementary Table 4.** The mediation effect of blood biomarkers on the association between statin use and global cognitive function among participants without history of coronary heart disease, stroke, diabetes and hypertension.

| **Mediator** | **n** | **Total effect**  β (CI) | **Direct effect**  β (CI) | **Indirect effect**  β (CI) | **Proportion mediated** | ***P* value of the mediated effect** |
| --- | --- | --- | --- | --- | --- | --- |
| LDL | 94028 | -0.096 (-0.175, -0.015) | -0.063 (-0.126, 0.004) | -0.033 (-0.049, -0.018) | 34.21 % | 0.01 |
| HDL | 85684 | -0.081 (-0.148, -0.013) | -0.079 (-0.143, -0.014) | -0.002 (-0.005, 0.001) | 2.43 % | 0.148 |
| Triglycerides | 94160 | -0.096 (-0.162, -0.03) | -0.097 (-0.162, -0.032) | 0.001 (2*10^-4^, 0.002) | -1.21 % | 0.024 |
| Blood glucose | 85614 | -0.082 (-0.147, -0.019) | -0.081 (-0.145, -0.02) | -0.001 (-0.003, 0.001) | 0.76 % | 0.536 |
| CRP | 94041 | -0.095 (-0.159, -0.03) | -0.10 (-0.163, -0.038) | 0.006 (0.004, 0.008) | -5.94 % | 0.002 |
| Vitamin D | 89593 | -0.097 (-0.165, -0.035) | -0.097 (-0.163, -0.035) | -5*10^-4^ (-0.002, 0.001) | 0.48 % | 0.43 |

**Supplementary Table 5.** The mediation effect of blood biomarkers on the association between simvastatin use and global cognitive function.

| **Mediator** | **n** | **Total effect**  β (CI) | **Direct effect**  β (CI) | **Indirect effect**  β (CI) | **Proportion mediated** | ***P* value of the mediated effect** |
| --- | --- | --- | --- | --- | --- | --- |
| LDL | 125865 | -0.074 (-0.135, -0.008) | -0.043 (-0.092, 0.011) | -0.031 (-0.043, -0.02) | 41.33 % | 0.016 |
| HDL | 114546 | -0.069 (-0.12, -0.013) | -0.067 (-0.116, -0.013) | -0.002 (-0.004, 0) | 2.82 % | 0.056 |
| Triglycerides | 125766 | -0.079 (-0.131, -0.026) | -0.079 (-0.131, -0.027) | 0 (-0.001, 0) | 0 % | 0.996 |
| Blood glucose | 114437 | -0.07 (-0.128, -0.018) | -0.069 (-0.125, -0.018) | -0.001 (-0.003, 0) | 1.94 % | 0.024 |
| CRP | 125597 | -0.078 (-0.13, -0.025) | -0.085 (-0.135, -0.034) | 0.007 (0.005, 0.009) | -8.60 % | 0.004 |
| Vitamin D | 120073 | -0.08 (-0.135, -0.023) | -0.078 (-0.132, -0.023) | -0.002 (-0.003, 0) | 1.93 % | 0.008 |

Abbreviations: CI, Confidence Interval; CRP, C-Reactive Protein; HDL, High-Density Lipoprotein; LDL, Low-Density Lipoprotein.

**Supplementary References**

1. UK Biobank. Touch-screen Pairs memory test. https://biobank.ndph.ox.ac.uk/showcase/ukb/docs/Pairs.pdf. Published 2013. Accessed April 26, 2022.

2. UK Biobank. Touch-screen Fluid intelligence test. https://biobank.ndph.ox.ac.uk/showcase/ukb/docs/Fluidintelligence.pdf. Published 2012. Accessed April 26, 2022.

3. UK Biobank. Touch-screen test of reaction time (Snap). https://biobank.ndph.ox.ac.uk/showcase/ukb/docs/Snap.pdf. Published 2015. Accessed April 26, 2022.

4. UK Biobank. Touch screen test of Prospective memory (Shape). https://biobank.ndph.ox.ac.uk/showcase/ukb/docs/Shape.pdf. Published 2012. Accessed April 26, 2022.
